# Cripping inquiry: breathing life into co-produced disability methodologies

**DOI:** 10.3389/fsoc.2025.1600693

**Published:** 2025-06-26

**Authors:** Julie Ellis, Louise Atkinson, Suzanne Glover, Jennifer Kettle, Grace Joseph, Jamie Hale, Amanda Jones, Mitch Coles, Libby Bligh, Ruth Bridgens, Conor O'Kane, Jenny Negus, Haffizah Ali, Connor Thompson, Sarah Waters, Casey Coats, Barry J. Gibson, Kate Weiner, Rod Lawson, Kirsty Liddiard

**Affiliations:** ^1^School of Education, The University of Sheffield, Sheffield, United Kingdom; ^2^School of Clinical Dentistry, The University of Sheffield, Sheffield, United Kingdom; ^3^School of Sociological Studies, Politics and International Relations, The University of Sheffield, Sheffield, United Kingdom; ^4^Sheffield Teaching Hospitals NHS Foundation Trust, Sheffield, United Kingdom

**Keywords:** co-production, crip, ventilation, illness, care, embodiment

## Abstract

**Introduction:**

Our contributions within this article emerge from our experiences of co-leading a new Wellcome Discovery Award funded project, *Cripping Breath: Towards a New Cultural Politics of Respiration*. As a diverse team of clinicians, artists, academics and others with lived and embodied experience of disability, chronic illness, and neurodivergence, we are broadly exploring breathing and ventilation (e.g., forms of medical technology that support respiration) through arts-informed, archival, narrative and ethnographic research approaches.

**Methods:**

Cripping Breath aims to forge new understandings of respiration from crip perspectives, which unapologetically center disability as a valued human experience. In this article, we unpack the meanings, politics and practices of crip perspectives and methodologies - forms of knowledge production that emerge from lived and embodied experiences of disability and chronic illness - and consider their contributions to our project so far. We think through crip time, Slow scholarship and (seemingly) radical things like rest and recuperation, and grief and loss within the research process.

**Results:**

We share the importance of embracing flexibility, adaptability and radical care as routine across our team, because we all bring various types of impairment, embodiment, chronic illness, and caring responsibilities.

**Discussion:**

We question the meanings of these forms of *welcoming in* disability, impairment and difference as ways to develop radical and cripcultures of co-produced and innovative disability research methodologies, and conclude by calling for a more inclusive sociology.

## Introduction

Cripping Breath: Toward a New Cultural Politics of Respiration is a 5 year transdisciplinary program of research funded by the Wellcome Trust. It centers and explores the lives of people who have had their lives saved and sustained by ventilatory medical technologies. Centring arts-informed, archival, narrative and ethnographic approaches, Cripping Breath develops crip perspectives - forms of knowledge production that emerge from lived and embodied experiences of disability and chronic illness. Academics, researchers, experts-by-experience, clinicians and artists are working in collaboration to co-curate and co-produce new understandings of the experiences of ventilated people, across a host of identity positions, to interrogate the new cultural politics of respiration and ventilation in a continuing global pandemic, and as we imagine post-pandemic futures. Cripping Breath centers a range of methodological approaches to explore the experiences and meanings of living on forms of ventilation. To clarify, when we talk about ventilation, we are referring to non-invasive forms of ventilation such as Continuous Positive Airway Pressure (CPAP) or Bilevel Positive Airway Pressure (BIPAP) technologies; but our project is also seeking to explore invasive forms of ventilation, such as a tracheostomy or intubation during times of (respiratory) crisis. Our focus on ventilatory technologies pulls into view a range of people from different kinds of impairment and illness categories: people with neuromuscular impairments and associated respiratory illness; people with acquired respiratory illnesses (such as Chronic obstructive pulmonary disease: COPD); people with respiratory failure; and people who may encounter ventilation on the trajectory of other forms of progressive illness, such as Motor Neurone Disease (MND). We want to know: (i) what new forms of scholarship are needed to radically transform understandings of respiration and ventilation; (ii) how we can better understand the social, cultural, political and material meanings of ventilation and breathing during an ongoing global pandemic, and as we imagine post-pandemic futures; and (iii) whether we can propose the ventilatory experience as a space to promote new conversations about life, death, disability and health. Further, we want to know, (iv) about the kinds of affective, relational and intimate relations that may be engendered in and with the medical technologies that sustain and save the lives of ventilated people; (v) the ways in which the creative artistic process can authentically capture the realities of living with ventilation; and across inquiry, as a co-produced project, we want to (vi) better understand what kinds of principles and practices of co-production need to be developed to enhance health-related research.

Such a range of questions demands a transdisciplinary and multi-methods approach, and we outline these here. In order to explore the ways in which creative processes can capture the realities of living on and/or with ventilation, in our *Arts Stream* we employ performance (theater-based) and contemporary art practice, led by two Artists-in-Residence (Hale and Atkinson) to the project, to animate and give form to breathing and respiration as elements of life and death that are invisible, formless and taken for granted. Our Artists-in-Residence are people with lived experiences of both ventilation, progressive physical impairment and/or neurodivergence. The arts have long been used to examine the significance of breath. According to Fahd (2019: 177) ‘…while breathing operates at the margins of perception, its symbolic possibilities are frequently visualized in photography, video and performance-based works’ (see also Tremblay, 2018). We propose that accessible creative processes will offer new social texts of respiratory health and illness which can be the very means through which ‘to draw attention to the unobserved role of the breath in everyday life’ (Fahd, 2019: 177). Furthermore, research-informed theater has become a powerful tool to share research and co-construct data in radical ways that ‘disrupt hegemony while offering a platform for counter hegemonic narratives and doings to appear’ (Schott, 2021: 117). We label our arts-methods participants as collaborators in recognition that they are also key knowledge producers in the process; as such our Artist Collaborators (ventilated people) are being remunerated as artists and will co-lead curation, exhibition and performance.

In order to make space for disabled, chronically ill and ventilated people to speak with and back to respiratory physicians and health services, communicating the lived and embodied experiences of ventilated lives, our *Ethnographic Stream* applies collaborative and creative ethnographic approaches specifically to patient ventilation journeys as these are happening in real-time. Our approach will involve spending time in hospital (the Northern General in Sheffield) observing clinicians at work and discussing their practice in interviews to understand the respiratory culture within which patients negotiate their treatment and seek support. We will also spend time with patients over a period of around 6 months to understand how they experience their initial diagnosis and intervention, and how they adjust to living on and with ventilatory technologies in the longer-term. Thus we will specifically explore instituting, or beginning, ventilation as a health intervention; its relationship to issues of ‘patient compliance’; the temporal and negotiated understandings of quality of life and ventilated futures and the ‘activity spaces’ regarding ventilator use (Walker et al., 2020). During these 6 months with participants we anticipate that we will encounter significant others too - relatives, friends, personal assistants, neighbors, pets - whose perspectives might help us to further understand the relational dimensions of living with ventilation. We will use a variety of ethnographic approaches including interviews, observation, video diaries and creative scrapbooking to offer participants choice in how they would like us to ‘be’ with them over this time. Our aim will be to co-construct with participants an approach which allows us to ‘follow’ ventilatory and breathing technologies from respiratory clinics at the hospital to participants’ homes to explore clinicians’ and patients’ understandings, expectations and negotiations of ventilator practice over time. Taking radical inspiration from a new ‘patchwork’ approach to ethnographic research (Günel et al., 2020), in a later section of this article we consider what ‘gentle’ co-construction in ethnographic research might look like - acknowledging that the personal commitments, priorities and needs of researchers are also an important consideration in care-full (see Budworth, 2023; Lonkila, 2021) research design.

Our *Narrative Stream* is being led by our Co-researcher Co-operative, a group of experts-by-experience that are employing virtual narrative methods to capture participants’ stories of ventilation. Often non-academic co-researchers are included in inquiry only in tokenistic ways; for example, they may be routinely excluded from the parts of the research that are deemed the preserve of academics, such as analysis and publication (Liddiard et al., 2019). To counter this, narrative and Photovoice data collection and analyses throughout the project will be accessible, collaborative and co-led by the Community Researcher Co-operative, who are being paid, and formally employed by our institution, as researchers across the project. Beyond research design and data collection, community researchers will co-lead public engagement and dissemination across multiple contexts, and will co-author for publication (see Liddiard et al., 2022). We discuss the value of the Community Researcher Co-operative to innovative disability research methodologies later in this article.

Finally, to consider the ways in which ventilation can be a vehicle for new conversations about life, death, disability and health in an ongoing pandemic and as we look to post-pandemic futures, in the *Archival Stream* we are currently virtually exploring sources that relate to both respiratory health and illness and ventilation as medical intervention and treatment. Embodying the politics of rewriting histories from the perspectives of marginalized people, we are re-conceptualizing archives to understand respiratory health in new ways in the context of archival sociology (White, 2012; Benzecry et al., 2020). We aim to ‘radicalize traditional approaches’ (Brilmyer, 2018: 1) in order to shift power relations that are historically reproduced through archives.

Now we have outlined the shape of our research design, in the next section of this article we critically reflect upon our first year working as a team to *crip* inquiry. We understand crip to mean ‘the non-compliant, anti-assimilationist position that disability is a desirable part of the world’ (Hamraie and Fritsch, 2019: 2). For us, crip extends to the research process, which includes how we support and care for each other to manage our project as a team. In doing so, we discuss our imperatives to embed inclusive working practices, develop relationships, and design care-full (see Budworth, 2023; Lonkila, 2021) methodological approaches. Following this, we move on to reflect upon the meanings and implications of rest and self-care as routine aspects of our research process - also an act of Crip. In the final section, to ‘build on a growing tradition of sharing the challenging moments of qualitative research’ (Bowtell et al., 2013: 652), we explore our own recent experiences of death within the research process and consider the meanings of grief, loss and legacy in both disability research and sociologies of health and illness. We conclude by calling for a more inclusive sociology.

## Cripping inquiry: the story of our first year

In Cripping Breath, we are very purposefully engaging in slow scholarship (Mountz et al., 2015) to counter forms of ableism experienced by disabled researchers (and others) within the academy, to create an environment in which disability is desired and vital. We define ableism as ‘an ideology that privileges able-bodiedness and -mindedness and a preferential citizen as self-sufficient, autonomous, independent and entrepreneurial’ (Goodley et al., 2025: 121). To desire disability is underpinned by our understanding of crip, which is also informed by McRuer (2006: 35), with crip being that which ‘questions or takes a sledgehammer to that which has been concretised’. Our project title - Cripping Breath - demarcates our desire to unsettle, to contest and challenge normalcy around breath and breathing. We are also conscious of doing this work as a team made up of disabled, ventilated, chronically ill and ally researchers. Liddiard and Lawthom (in press: np) state that ‘critical to the way in which disabled women (and others) theorize disability in the world inherently relates to the conditions of an ableist academy’. As Jain (2023: 30) maintains, ‘the university is deeply rooted in ableist practices’. As Goodley et al. (2025: 121) explain: ‘disabled students and staff experience exclusionary admissions and recruitment, poor career pipelines and in/formal support, under-employment and precarity’. Brown and Leigh’s (2020) excellent edited collection of writings has for the first time emphasized the ways in which disabled and chronically ill academics and researchers are now more cognisant of the ableist environments in which we work and the ways in which certain types of bodies and minds are both unexpected and unwelcome in the academy (see also Brown and Leigh, 2018). Aptly, ‘hiding, keeping up, disclosing, pushing yourself, coping, passing and masking are all practices that require emotional and other forms of labor for disabled and chronically ill people, both inside and outside of the academy’ (Liddiard and Lawthom, in press: np). Wilkinson and Wilkinson’s (2023: 4) powerful writing articulates the ways in which sick, disabled and ill bodies and people have to labor to ‘perform (un)spoiled academic identities’.

One of our project responses to such academic ableism, then, is slow scholarship, which ‘questions the ever-increasing demands of academic life, placing them broadly within wider tendencies toward neoliberal university governance’ (Mountz et al., 2015, p. 1238). As some of us have said elsewhere, it ‘…involves resistance, engaging slowly with the object of study, engaging with others and improving the quality of academic practices such as writing’ (Liddiard and Lawthom, in press: np). For us currently it involves playing with and pushing at the normative temporalities of the research process; often in ways that better fits a diversity of identities, embodiments and experiences. But it also relates to the ethics of how we wish to work. In Cripping Breath it began at the stage of co-designing the project in preparation for the funding bid to the Wellcome Trust. Cognisant of the ways in which authentic forms of co-production with marginalized people and their communities demand an ethic of care, we wanted to enact a co-production process in our project that meant something to disabled, chronically ill and ventilated people. A process that centers relational labors and a crip and feminist ethic of care which involves ‘empathy, reflection, anticipation, affirmation and compassion’ (Katzman et al., 2020: 519). Important to us all was that we took our time, and never felt rushed; accounted for illness, vulnerability, ableism and care within the process; and understood co-production, first and foremost, as a deeply relational practice (see Liddiard et al., 2024). Importantly, to support this, we applied for and were successful in gaining seed funding to pay partners and collaborators to support the co-authoring of the project application for the funder (see Liddiard et al., 2024). To be more transparent here in our aim to support other researchers, our principal investigator accessed a Women Academics’ returning to work Program (WARP) at our institution. This fund is purposeful toward supporting women academics in their return to research following periods of parental leave. We recognize the privilege of access to such internal funding, and that part of this is from being in a research-intensive university in the UK context.

Together, we co-designed and advocated for a 5 year project (2024–2028). This allowed us a full first year to come together as a diverse core research team - we are clinicians, artists, academics and others with lived and embodied experience of disability, chronic illness, and neurodivergence. Such transdisciplinarity means careful work, to listen to and appreciate one others’ perspectives and enact access in considered ways. Furthermore, in year one, we recruited our research associates through an inclusive process layered with care. Elsewhere we have articulated what this involved: ‘removing ableist language from job descriptions and person specifications; flexible forms of interview; accessible inductions and onboarding; and flexible and remote working as routine’ (Liddiard et al., 2024: 13–14). We had feedback from interviewees (who did not get the posts) which said that they were grateful for a “fully humanized process” (personal correspondence, 2024) that was inclusive, accessible and - despite the institutional and bureaucratic context of university recruitment (see Goodley et al., 2025) - *caring*.

Furthermore, we have spent our first year deeply exploring the contexts in which we want to collect data: disability arts cultures and contexts and visits to see and build relationships with our disability organization and arts partners; visits to hospitals and healthcare spaces to learn the cultures in which our clinical ethnography will take place; sitting in (virtual) archives exploring the histories of medical technologies; and collectively thinking through inclusive and creative approaches to how we wanted to recruit our community researchers. Key to our co-produced approach is our Community Researcher Cooperative - a team of community-based researchers - all of whom live on and with ventilatory technologies and respiratory illness, who are working across the project to embed lived and embodied knowledge into our theory-building and co-lead our inclusive approaches to inquiry (see Liddiard et al., 2023). In our first year we have recruited 13 diverse community researchers and employed everyone on university employment contracts. This in itself was a process - beginning with liaising with Human Resources (HR) and other university systems about flexible and small contracts (e.g., contracts of 2–3 h per week) as well as advocating for grade 7 pay (a UK postdoctoral pay level) for people who are from a range of educational and employment backgrounds. This took an extensive amount of labor and negotiation with HR and others to understand why community researchers were working on such small fractional contracts; we hit institutional barriers here in terms of what labor looks like in the academy, and who is expected to be doing it (see Goodley et al., 2025 for a discussion of university bureaucracy as it relates to research processes). Our advice for other researchers here is to persevere - changing standardized institutional understandings of ‘contribution’ and ‘labor’ takes: (i) lots of time; (ii) collaboration *with* university systems and processes (rather than working against them); and (iii) the support of our Professional Services colleagues whose work sits within these systems. A good example of the latter here is that the technology the university uses to do the required Right to Work checks in the UK for someone to be eligible for employment was not accessible to many of our community researchers. This took significant support from our School Operations Team, and the kindness of a key Professional Services colleague here, to support community researchers to do manual Right to Work checks in order to become formally employed in contracts. Another hurdle we faced - not in relation to the institution - was ensuring that community researchers’ income from government benefits were not impacted by their work on the project. We had to very carefully - and individually - work with each community researcher who was a recipient of benefits (‘welfare’) to work out how to manage university pay and income in a context where certain governmental benefits only allow permitted work hour/pay limits.

Moving forward, we continued on and developed an accessible recruitment animation with British Sign Language and Easy Read applications (Glover, 2024)^1^, followed by accessible online interviews, and a program of work which commenced in early 2025 (year two) that centers things like co-authoring a collaboration agreement, learning about narrative research together, and undertaking a collaborative institutional ethical application process.

Taking our time with the recruitment of our community researchers has meant that they are beautifully diverse: aged from 18 to 60+ with varied experiences of ventilatory technologies, some with a tracheostomy, others who use forms of non-invasive ventilation (NIV), some for a few months, others for a lifetime. People with congenital and acquired respiratory illness and impairment; people with myriad life experiences - former and current NHS workers, charity trustees, activists and campaigners, artists, volunteers and advocates and more. All community researchers share lived experiences of ventilation as an intervention in their lives and have a passion for social research and learning about the experiences of others. These were our only eligibility criteria.

The space for community researchers to explore and co-create on the project is organic to the often-changing needs and skills of the team. The Co-operative encourages flexible working patterns and aims to dismantle neoliberal-able (see Goodley, 2014) needs for consistency and routine. Instead, the Co-operative facilitates a space that enables fluctuating work patterns around other commitments, periods of ill-health and simply harnessing windows of “good health” to ‘live’. Practically this is implemented with online meetings being recorded and made available on shared workspaces, asynchronous working, and 10 weeks of community researcher training that can be completed over 6 months. The space in which experiences are shared, and ideas are generated, is not bound by specific means of contribution, such as written feedback, but instead open to input in ways most comfortable to the community researcher including one-to-one informal conversations, group messaging and short and discreet reflexive tasks. The Lead Community Researcher (Glover) and Research Associate (Kettle) work closely to weave and bring together ideas into a shared vision for the Co-operative. Importantly, we designed a specific project post around supporting community researchers (the Lead Community Researcher) through learning about the everyday labors in making conventional research processes accessible to those who have not had formal academic training in a former project (see Liddiard et al., 2022).

Similarly, in the Arts Stream, we are currently in the process of recruiting six disabled artist collaborators who will undertake paid research informed theater and contemporary arts residencies within the project. Led by our Research Associate (Joseph) in collaboration with our Artists-in-Residence, this again is careful and critical work that rightly takes *time*: What counts as an artist, and art? Who do we need or want to work with? What kinds of ventilatory experiences do we require here? What counts as ventilation? How can we develop asynchronous, virtual, and accessible ways of making art and theater together? Meetings are (often joyfully) spent discussing, imagining, and thinking. We have had to undertake multiple complicated institutional ethics applications to enable this work to move forward; and our recruitment processes are being carefully curated and ‘translated’ into accessible formats such as British Sign Language (BSL) and Easy Read. We have had to reflect on *how* we will recruit our six artist collaborators, and put a lot of thought into the politics and practicalities of *selection*; and most importantly, the ethical considerations to support those who are *not* selected for a residency.

Thus, rather than jumping into data collection or systematic literature reviews as often happens in the first year of a funded empirical research project, we have spent time together, learning and exploring together, thinking critically about how we will collaborate and co-produce new knowledge together. Viney explores the “projectification” of academic research: ‘It interests me that projects attempt to resolve research aims, questions, collaborating organizations, methods, and outputs before beginning their work. In this sense they are an organizational form antithetical to discovery research’ (Viney, 2024: np). Moreover, ‘…In the economic life of the project human lives – contract workers, participants, ‘patients’ – are rendered as technical inputs and outputs, so the performance of projects can be measured, graded, and optimized’ (Viney, 2024: np). Thus while we have had plans for our first year (because our funder required these as a prerequisite for funding), these have had to be truly flexible, moveable and subject to change at any time. As we have said elsewhere, Cripping Breath seeks to ‘push the boundaries of what’s possible (or not) in the neoliberal academy to play with the temporalities of normative research processes which are typically fast-paced, metric and output-oriented, inaccessible to many (and thus exclusionary), and which are fixed to accelerated timelines and follow the temporal regimes of the neoliberal university’ (Liddiard et al., 2024: 11).

## Rest, recuperation and care

This section of our article follows on from an initial piece of writing published last year in the online medical humanities journal, *Polyphony* (Atkinson et al., 2024). In this short piece, some members of the research team came together to ‘draw upon personal narratives and embodied experiences of respiratory failure and neurodivergence to think through crip time’ (Atkinson et al., 2024, np). Working with Kafer’s (2013: 27) understanding of crip time as ‘flex time not just expanded but exploded’ we explored the ways in which time is experienced in different contexts and by different people within the wider project team, to understand how our inquiry can meaningfully center disability experiences, caring and embodiment. We also follow White (2023: 5), who defines crip time as ‘…a flexibility and an expansion of time, both in response to bodily necessity and to societal barriers that make it so that more time may in fact be necessary.’ It was through thinking what time is and means, as humans working across a transdisciplinary research project together, that we arrived at the importance of rest, recuperation and recovery time in a project about health and illness led by disabled, chronically ill and neurodivergent researchers. In short, our desire for rest, recuperation and recovery time in Cripping Breath is a necessity, and we are making space for it and want to feel safe as we do so. In just 1 year of our project, we have had multiple team members die, be hospitalized, undergo emergency surgery, routinely be ‘off sick’ and need time away from the project (for themselves and those they care for), and we have had COVID-19, chest infections, pneumonia and influenza multiple times. As we have reflected previously, ‘living with forms of respiratory impairment and/or using ventilation can mean dealing with fatigue, breathlessness, limited energy (particularly over longer periods of time), and a sensitivity to minor illness, whereby something as simple as catching a cold can mean weeks of struggle and recovery’ (Atkinson et al., 2024: np). Thus, we are a project of vulnerable bodies (our own and others whom we care for). On the project start date, our Principal Investigator was sitting in an acute respiratory ward in hospital just focusing on trying to keep breathing - the irony was palpable - but this is what Cripping Breath seeks to be: inquiry that centers lived and embodied experiences of respiratory illness, often in the rawest of ways. This can have very real material realities for our ways of working (Atkinson et al., 2024: np):

‘Project processes can and do get slowed down by prioritising flexibility around hospital appointments, taking time off sick, waiting for antibiotics and other medications to kick in, and managing sudden hospitalisations and surgeries. Actively making space for the team to rest, recuperate and recover takes on a new meaning as we build in contingencies, use organisational technologies to share and document our work so someone else can jump in when needed, and resist the work-intensive temporalities of academia’.

As we enter year two of our project, then, we are working on ways of co-developing a research environment and project culture that gives foundation to easier conversations about asking for ‘time away’ - in a way that does not ignite our own internalized ableism. Similar to internalized oppression, which ‘results in group members loathing themselves, disliking others in their group, and blaming themselves for the oppression’ (Rosenwasser, 2000: 1), internalized ableism operates as a form of psycho-emotional disablism. Psycho-emotional disablism is defined by Thomas (1999: 60) as ‘the socially engendered undermining of emotional well-being’. As Reeve (2004: 84, 2008) contends, for disabled people and others, it operates ‘at both the public and personal levels, affecting what people can do, as well as what they can be.’ As we intimated at the very beginning of this article, the university is a deeply ableist space - with ableism being ‘associated with the broader cultural logics of autonomy, self-sufficiency and independence’ (Goodley et al., 2018: 209). As disabled and ally researchers, then, to counter this, we are putting importance upon working in ways that encourage connection, care and interdependence as a team. In practice this means embracing flexibility, adaptability and radical care across the team, because we all bring various types of impairment, embodiment, chronic illness (see Piepzna-Samarasinha, 2018), as well as forms of caring responsibilities for intimate others. Explicit and unapologetic, or crip, recognition of this is both a political and practical matter. We are arranging our day-to-day work practices in ways which directly challenge narrow ableist notions of how we can be productive while harnessing the camaraderie which comes from experiencing ‘vulnerability’ as something we do not need to mask, ‘power through’ or feel ashamed about.

It is possible to center rest, recuperation and care within research design too, which is something we have taken our time to consider when setting up the ethnographic stream of Cripping Breath. Ethnography as it is traditionally (anthropologically) understood conjures up an image of the lone (often male, nearly always able bodied) researcher who is completely immersed in a distant, unfamiliar place for months, maybe even years, at a time. While there have been many challenges to this outdated, colonialist vision of ethnography (Uddin, 2011), oftentimes scholars assert the importance of doing ‘ethnography at home’ (Anderson, 2021) or they argue that contemporary social contexts require something different from ethnographers (e.g., online ethnography). While we completely agree with these arguments, the notion that ethnographic researchers *themselves* might need things that shape the kind of ethnography and knowledge production that is possible, necessitates rather more radical thinking. In our work we have been influenced by a rallying call for ‘patchwork ethnography’; ‘a new methodological and theoretical approach’ which not only advocates for spatial and temporal reconfigurations of what it means to be ‘in the field’ but which also calls out the ableism and depoliticisation of researcher positionality that underpins traditional ideas about ethnography (Günel et al., 2020: np). A patchwork approach values the entwining personal and professional aspects of researchers’ lives and the possibilities this generates for ‘innovating methods and epistemologies to contend with intimate, personal, political, or material concerns’ (Günel et al., 2020: np). Put simply, the personal, relational and embodied needs of researchers and those they care for are important considerations in planning the logistics of how ethnographic research gets done. But more than this, these considerations make for productive, ‘kinder and gentler ways to do research’ (2020:np). As the authors write (Günel et al., 2020: np):

‘Rather than see the multiple commitments of researchers as constraints, we will reflect on what forms of knowledge and methodologies emerge in and through researchers’ life and work commitments’.

Leaving the field to collect a child from school, taking an hour away to attend a medical appointment with a partner, scheduling observations and interviews so there is time to get lunch, to go for a walk, to collect a prescription - by insisting these examples of everyday care are methodological issues, we are ‘cripping’ ethnographic design in ways which feel both deeply mundane and yet powerfully radical. In our ethnographic work which is being led by Ellis - a mother with a young child and caring responsibilities - we are actively planning fieldwork around regular days off and school holidays. We are also thinking about ways to avoid debilitating feelings of overwhelm which we know from personal experience can develop from feeling overstretched and internalizing ableist and neoliberal ideas about personal responsibility, professionalism and labor. Thus we argue that to configure the logistics of research by leading explicitly with compassion, and acknowledging the often complicated, messy, demanding lives of *both* participant and researcher is an important way to center inclusivity, recuperation and care in any sociological research design. As we shall explore in the next section on loss, navigating the fragility and unpredictability ever present in disability research (Budworth, 2023) makes this especially important.

## Disability and death: accounting for loss in the research process

*“We’ve got to start talking about death and dying. We need to reclaim the language. We need to narrate dying. It’s time.”* (Watts, 2018)

The words above were written by a former colleague, Lucy Watts MBE, who has since died. Lucy worked with some of us as a co-researcher in a former project (see Liddiard et al., 2023 for the full story of Lucy’s contributions), and much of her work was rooted in promoting the need for and improving end of life planning and palliative care for young people (Watts, 2021). Her words here echo our desire to think carefully and mindfully about death in our project. Just days after Cripping Breath began, our key collaborator Sally Whitney-Mitchell - a brilliant researcher who co-designed much of our co-production approach - lost her life at just 36. Sally was a researcher who enacted an ethic of care like never before. A researcher without any formal training, someone who fell in love with inquiry and writing (Whitney et al., 2019; see also Evans and Whitney-Mitchell, 2023) in her role as a lived experience co-researcher in a former funded project about disabled young people living with life limiting and life threatening impairments (Goodley et al., 2018), Sally was the lynchpin in our relational and affective approaches to co-production. Sally’s sudden death understandably took a lot of time to come to terms with for us as a team. Some of us had been working with Sally, across projects, for a number of years prior to her death. Since Sally’s death, we have also lost two of our community researchers. Their deaths occurred suddenly and very soon into their time working on the project. Beyond the sadness, a number of responsibilities came into view quickly: How do we inform our other community researchers, and what support could/should we offer when we do? How do we mark late community researchers’ contributions to the project? What, if anything, should we “say” as a project publicly? How can we send our love and best wishes to their partners, family members, and communities (see Bowtell et al., 2013)? Research that takes place in institutions (in this case, the university) also causes and demands a bureaucratic response (see Goodley et al., 2025). Things like reporting the death of a staff member (because our community researchers are on university contracts); negotiating with HR about the termination of contracts (‘I can confirm that no further action is needed for the post. We can pick this up again when you are ready to re-recruit’); and having difficult conversations around outstanding pay. Crip time is a useful lens here to understand the institution as needing to sequester death away quickly and neatly (see Samuels, 2017).

In this section, we focus on grief, loss, care and legacy in the research process in order to ‘build on a growing tradition of sharing the challenging moments of qualitative research’ (Bowtell et al., 2013: 652). We also again locate grief and death in the context of crip time; as Ellen Samuels says, ‘Crip time is grief time’ (Samuels, 2017; np). Our transparency - we hope - is productive toward supporting other researchers and projects. As a caveat, though, as people aligned to disability studies, we also recognize the risks in associating disability, vulnerability and death - or the personal tragedy model of disability (see Goodley et al., 2018) - in global ableist cultures where disabled people and their families are fighting for rights to live, thrive, be educated, employed and be included in their communities. From Britain, where we are writing, disabled people’s communities are actively fighting against the Terminally Ill Adults (End of Life) Bill 2024–25 which has recently had its second reading in the House of Commons, and we are also currently witnessing a new wave of austerity - which (again) centers on “reducing the costs” of disabled and chronically ill people - from our newly elected labor government. We understand deeply, then, that these ableist times promote and enact forms of neo-eugenic cultures that devalue the lives of disabled people (see Rembis, 2009; Shildrick, 2008).

Such a loss - the deaths of three team members in just 1 year of our project - has urged us to critically reflect upon death and bereavement. The very fact that we are exploring ventilation, respiratory illness, and disability as people with very particular clinical vulnerabilities means we, perhaps, have an inevitable proximity to death that is worthy of attention. Markedly, there is a relative lack of focus about death in research in the literature, and more specifically, its impact on researchers (De Laine, 2000). As Borgstrom and Ellis (2020: 591) state, ‘…less attention has been paid to researcher vulnerability specifically and its methodological implications’ (see also Silverio et al., 2022). What happens when someone dies in the research process? How should we talk, think, and feel about death in a research project? What kinds of human, and humane, responses are needed? What forms of support do we have access to as researchers? These are all current questions we are working through in Cripping Breath. To us, these are important questions in the context of disability research, particularly that which aligns itself with the politics of crip (McRuer, 2006).

Our early experiences of loss in this project have brought into critical view the need for us all to think and talk explicitly about grief, loss and death in a research project (Lundquist and Husebo, 2020). Death is, and will be again, present in Cripping Breath, and we need the time and tools as a team to make sense of this and the emotionality it brings (Harrison, 2021; Samuels, 2017). It also requires us to think in flexible ways about who is ‘in’ the team - about the role of legacy within research (of Sally’s, and others’, indelible contributions). Crip time is further relevant to these questions of legacy because of the ways it challenges normative ideas around time, bodies, and lifespans, and specifically, the finality of death (see Ljuslinder et al., 2020). We must also ask important questions about how we talk and/or connect with one another about loss mindfully and with care for the individual (health) circumstances of different members of the team. We are, quite literally, working it out as we move through the project. For example, we have procured the services of a specialist grief practitioner to create a bespoke workshop for the research team. While not intended to ‘workshop our way out of’ dealing with death, we see the workshop as a start of a conversation, and space, in which we can sit with grief in the process. For our community researchers, who not only have lived experience that may mirror that of participants, but who have not done emotionally demanding or sensitive research (Dickson-Swift et al., 2009) before, we have co-developed a self-care protocol with and for them. This protocol exists as a live document which supports community researchers to identify and voice their distress in the process as researchers while also providing different kinds of routes to self-care. The protocol features detail on emotionally demanding research and acknowledging distress; the practicalities of how to support ourselves and others in the team and developing a self-care plan; how to set boundaries as a novice researcher; information about community researcher debriefs we want to offer community researchers after every narrative interview with a participant; and how to take a break from the project if needed, with an “I need to take a break” email template for community researchers to use to make having some time away easier. Our Lead Community Researcher also practices regular “check-ins,” and research workshops with and for community researchers in this first phase of the project seek to attend to emotion, vulnerability and reflexivity - ‘an explicit self-analysis of one’s own role in research’ (Borgstrom and Ellis, 2020: 592) - as a central part of our narrative inquiry. Moreover our community researchers are keen to co-develop a further protocol as to how we respond as a team of researchers - if and/or when - a participant dies between their participation in the project and the publication of findings. Key questions here might center on the ethics of re-telling late participants’ stories; the emotional work of analyzing data from people we know have died; and again how to recognize and memorialize the deaths of participants. Our participants may die ‘early’, or young; or before those with expected, *normal* lifespans - what Samuels (2017: np) calls ‘the sheltered space of normative time’. As Samuels’s (2017: np) echoes, ‘Crip time is time travel. Disability and illness have the power to extract us from linear, progressive time with its normative life stages and cast us into a wormhole of backward and forward acceleration, jerky stops and starts, tedious intervals and abrupt endings’. Therefore, we are conscious to work against normative trends in the social sciences whereby, for many reasons, particularly for ethics committees, ‘often researchers are expected to conceal, deny, or demonstrate how they will minimize their [own] vulnerability’ (Borgstrom and Ellis, 2020: 591). One of our researchers (Ellis), who has researched the everyday aspects of illness, death, dying and bereavement in previous projects (see Reed et al., 2023; Borgstrom and Ellis, 2017; Ellis, 2013), has challenged us not to see grief and loss as inevitably harmful, but that in our future practice we need to find comfort in ‘sitting with the sadness’.

To again draw in crip time - ‘the non-linear, unpredictable, ever-changing, or multiply enfolded temporalities of being disabled’ (Chazan, 2023: 1) - thus far we have experienced the death of team members as *rupture*. Processes stopped or slowed, and had to be redesigned; talking about loss took precedence over process; sadness halted the ability to theorize; and legacy - making links between late team members’ past contributions and the future of the project - has come to the fore (see Samuels, 2017 for an exploration of the ‘less appealing aspects of crip time’, 2017: np). Thus, we want to argue here that these early experiences show that research is far more than an empirically-driven, increasingly metricised, bureaucratic exercise. *Feeling* our way through challenging experiences such as loss offers painful but potentially productive opportunities to enact crip politics as research practice - to find spaces, temporalities and ways of working that center intimacy, connection and care. In doing so we will challenge normative ideas about how research should ‘feel’, where, when and how it should happen and who it can involve.

## Drawing some conclusions

In this article we have essentially storied the first year of our project, Cripping Breath: Toward a New Cultural Politics of Respiration, a 5 year transdisciplinary program of research funded by the Wellcome Trust. In doing so, we have drawn back the curtains on our processes as a diverse team, and discussed some common key challenges in disability research methodologies such as negotiating accessibility; challenging ableist and institutional notions of productivity; and co-creating inclusive methodological design. Our understanding of crip as ‘the non-compliant, anti-assimilationist position that disability is a desirable part of the world’ (Hamraie and Fritsch, 2019: 2) has anchored us to thinking critically about (normative) research processes; how we support and care for each other to manage our project as a team; the ways in which we embed inclusive working practices across the project; how we develop relationships both inside and outside of the research team; and design care-full (see Lonkila, 2021) methodological approaches like patchwork ethnography (Günel et al., 2020). We have also reflected upon the meanings and implications of rest and self-care as routine aspects of our research process - for us, an integral act of cripping ways of being and doing in the project - and explored our own recent experiences of death within the project. We have shared the importance of embracing flexibility, adaptability and radical care as routine across our team, because we all bring various types of impairment, embodiment, chronic illness (see Piepzna-Samarasinha, 2018), and caring responsibilities. In telling our story, then, we hope our (often messy) experiences can inform and support other researchers to ‘build on a growing tradition of sharing the challenging moments of qualitative research’ (Bowtell et al., 2013: 652). We suggest this transparency may be a key way to develop radical and crip cultures of co-produced and innovative disability research methodologies and can support a more inclusive sociology.

## Data Availability

The original contributions presented in the study are included in the article/supplementary material, further inquiries can be directed to the corresponding author.

## Footnotes

^1^ https://youtu.be/KhYFrL4Q_QY

## In memoriam

We dedicate this article to our colleague and friend, Sally Whitney-Mitchell. We miss you every day. We also dedicate this article to Stephen Deal and Seán Fitzsimons. We were so looking forward to working with you further, and are deeply grateful for your contributions and care for the project while working in Cripping Breath.
